# A microsurgical bifurcation rabbit model to investigate the effect of high-intensity focused ultrasound on aneurysms: a technical note

**DOI:** 10.1186/2050-5736-2-21

**Published:** 2014-10-31

**Authors:** Daniel Coluccia, Javier Fandino, Serge Marbacher, Salome Erhardt, Jenny Kienzler, Ernst Martin, Beat Werner

**Affiliations:** 1 Department of Neurosurgery, Kantonsspital Aarau, Tellstrasse, 5001 Aarau, Switzerland; 2 Center for MR Research, University Children’s Hospital, Zürich, Switzerland; 3 Cerebrovascular Research Laboratory, Department of Intensive Care Medicine, University of Bern and Bern University Hospital, Bern, Switzerland

**Keywords:** Focused ultrasound, Sonication, Aneurysm, Rabbit model

## Abstract

**Background:**

Recent clinical studies confirmed the high potential of MR-guided focused ultrasound (MRgFUS) in the field of functional neurosurgery. While its ability for precise thermo-ablation within soft tissue is widely recognized, the impact of high-intensity focused ultrasound (HIFU) on larger vessels is less explored. We used a bifurcation aneurysm model in rabbits to investigate the possible effects on the walls of vascular aneurysms and to assess the risk and prospect of this procedure for managing neurovascular disorders.

**Methods:**

Experimental bifurcation aneurysms were microsurgically created in New Zealand white rabbits and sonicated using MRgFUS.

**Results:**

A temperature of max. 54°C could be achieved close to the aneurysm, and the shape and size of the aneurysm were noticeably changed, as shown by MR angiography.

**Conclusions:**

The presented rabbit model proved suitable and capable of being extended to acquire data on the effect of HIFU on aneurysms and larger vessels. The fact that HIFU led to an alteration of the aneurysm without inducing rupture encourages further investigations.

## Introduction

High-intensity ultrasound focused into a small volume is known to generate heat, which enables precise and non-invasive thermo-coagulation of tissue [1]. Whereas the first attempts to apply this physical phenomenon in clinical use were troubled by a lack of thermometric control and exact guidance of the focal point, today it is possible to combine the delivery of ultrasonic beam with magnetic resonance image guidance, allowing thermometric monitoring and accurate targeting. Phased array transducers and CT-based phase correction allow compensation for wave aberration at the calvaria and targeting of intracranial regions selectively and precisely for ablation. Therefore, MR-guided focused ultrasound (MRgFUS) has been notably successful for non-invasive functional neurosurgery [2,3]. Focused ultrasound has been shown to be capable not only of ablating soft tissue but also of occluding arteries and controlling bleeding [4,5], presumably through a combination of a thermal coagulative effect on the vessel wall and mechanically induced blood flow stasis leading to thrombosis. In this technical note, we investigated the usefulness of a microsurgically produced bifurcation aneurysm rabbit model to study the influence and possible risks of MRgFUS on aneurysms in order to assess this innovative technique as an option for future neurovascular procedures.

## Methods

The study protocol was approved by the Swiss Animal Care and Experimentation Committee (approval number BE 14/10). All aneurysms were created in adult female New Zealand white rabbits (3–4 kg). A detailed description of the aneurysm creation is presented elsewhere [6]. In brief, general anesthesia was induced by administration of ketamine hydrochloride (Pfizer AG, Zurich, Switzerland) and xylazine hydrochloride (Vétoquinol AG, Ittigen, Switzerland) and continued intravenously.

### Aneurysm creation

**Figure 1:**
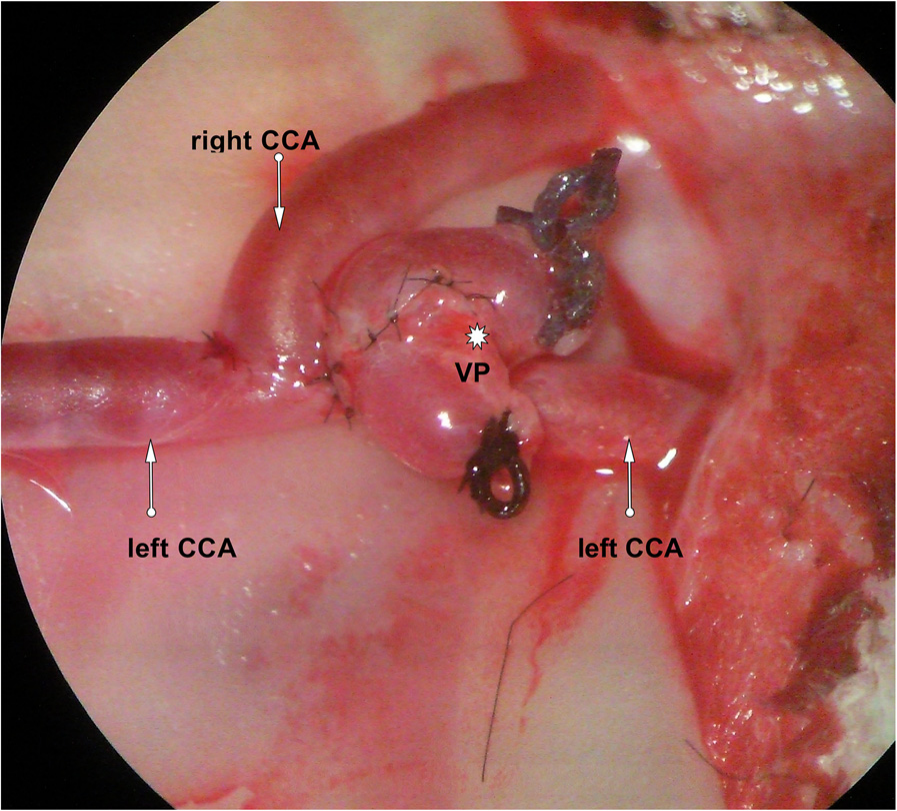
Microscope view of both the CCA (arrows) and the venous pouch (VP; star) after anastomosis resulting in an artificial bifurcation aneurysm.

Under an operating microscope, a 1-cm segment of the external jugular vein was harvested and used as a venous pouch. Both common carotid arteries (CCA) were prepared, and after ligation, the stump of the right CCA was mobilized to the left CCA. After an elliptic arteriotomy, the stump of the right CCA and the venous pouch were sutured to the left CCA using an end-to-side anastomosis with 10-0 thread (Ethilon, Ethicon Inc., Somerville, NJ, USA), resulting in an artificial bifurcation aneurysm (Figure 1). In order to allow epithelialization of the vessel walls, the MRgFUS treatment was carried out a minimum of 3 weeks after aneurysm creation. Furthermore, skin wound healing had to be advanced to a point where the stitches were completely absorbed and no obvious scar formations were detectable that could provoke additional acoustic aberrations.

### Magnetic resonance-guided focused ultrasound sonication

**Figure 2:**
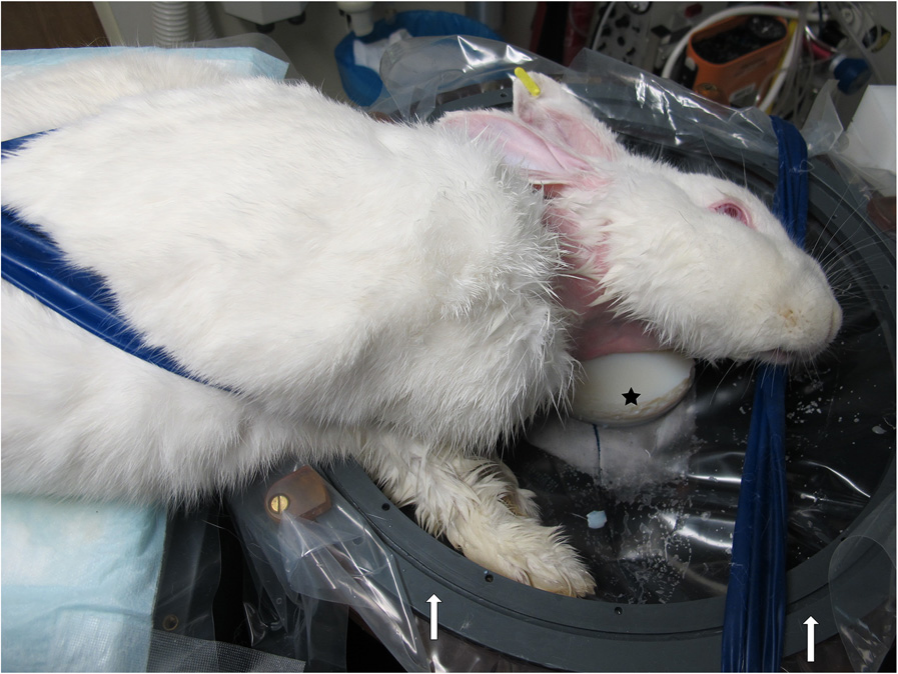
Rabbit positioning on the hemispheric transducer (arrows) using transparent film, elastic bandages, and gel blocks (star).

**Figure 3:**
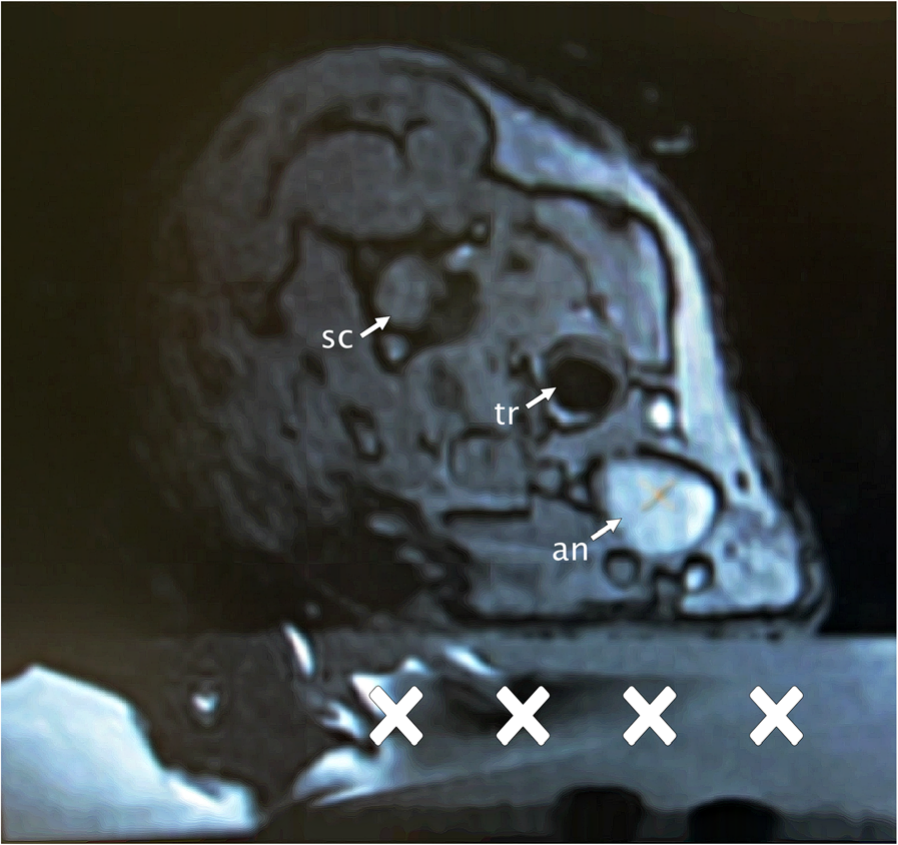
**MR angiography illustrating the aneurysm (an) and anatomical landmarks (tr = trachea, sc = spinal cord).** A supporting gel block (cross) allows correct positioning of the rabbits.

**Figure 4:**
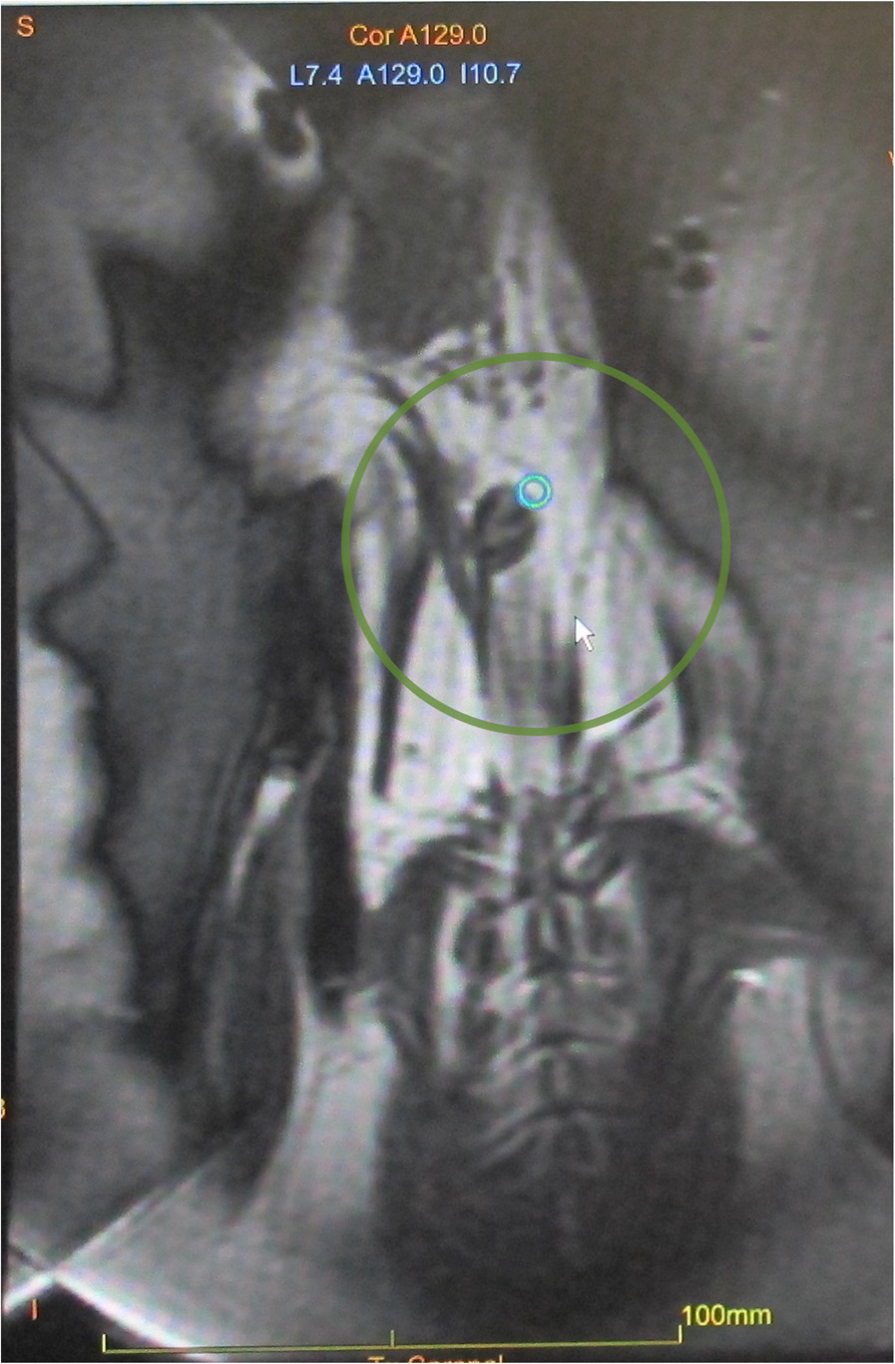
Sonication target (green circle) and effected tissue ablation of approximately 3 mm (blue circle) on the aneurysm.

The MRgFUS procedure was performed using the InSightec ExAblate 4000 system (Insightec Ltd., Haifa, Israel) integrated into a clinical 3T MR system (GE Healthcare, Milwaukee, WI, USA). The hemispheric phased array ultrasound transducer with 1,024 elements was driven at 650 kHz and oriented face up, filled with degassed water, and covered with a transparent film. After hair removal, the neck of the anesthetized rabbit was positioned on the film such that the aneurysm was aligned with the acoustic focus of the transducer. Tape was used to facilitate appropriate positioning and to stabilize the animal torso (Figures 2 and 3). In order to prevent the animals from aspirating water, acoustically transparent gel blocks supported the head. Liquid ultrasound gel and degassed water served as acoustic coupling media between the film and the skin of the animals. In the last two animals, the skin was opened through an incision to avoid sonication through the skin/fat layer. A clinical 7.5-cm surface coil (GE Healthcare, Milwaukee, WI, USA) was used for MR imaging. A flow-sensitive time-of-flight SPGR MR sequence was used to visualize the aneurysm before and after sonication. For MR angiography, a contrast agent (Omniscan, GE Healthcare, Milwaukee, WI, USA) was administered through a catheter in the ear vein. MR thermometry was based on phase-sensitive GRE sequences to evaluate the thermally induced proton resonance frequency shift. This method is known to be prone to movement and susceptibility artifacts. Since thermal measurements were not possible within the vessel or the vessel wall itself, readings were taken in stationary tissue close to the vessel. Estimating from the spectral Doppler velocimetry (SDV) in relevant regions of the phase images, uncertainty of thermal measurements under optimal conditions was <1°C. However, animal movement due to heartbeat and respiration as well as blood flow in the region of interest might have degraded measurement precision. Sonications were targeted at the border of the aneurysm wall (Figure 4). The focal spot of the ExAblate system used here has a full width at half maximum (FWHM) of 5–6 mm, which is sufficient to cover the vessel lumen. In order to facilitate thermal measurements, sonications were not always centered fully into the vessel but were also used to heat tissue adjacent to the vessel to obtain thermal readings from stationary tissue. For each animal, sonications started at 25-W acoustic power and 10-s duration. From there, the total acoustic energy was incrementally increased until temperatures above 50°C or an effect on the vessels such as alteration of shape or size could be detected by MR thermometry or angiography, respectively.

## Results

**Figure 5:**
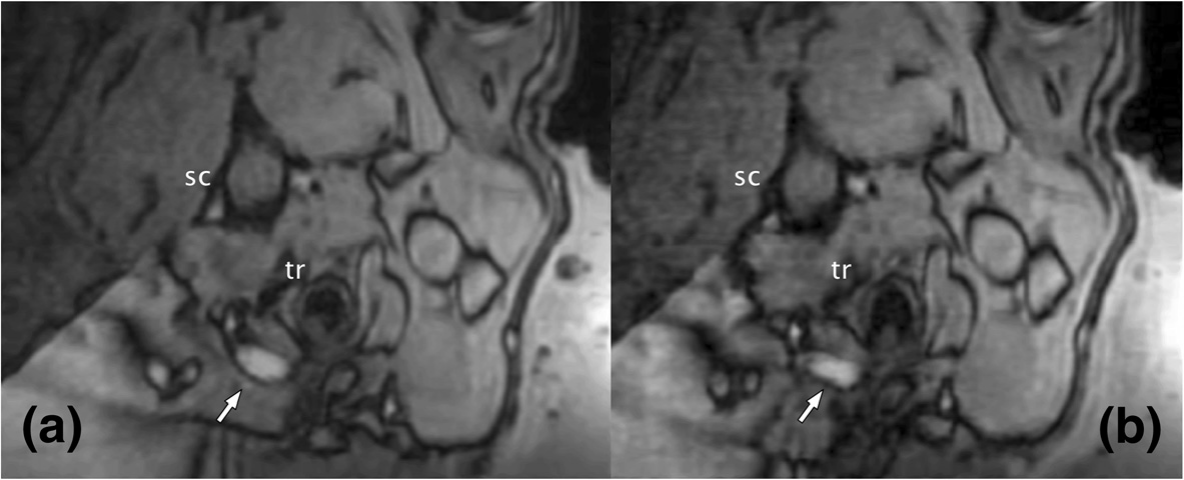
MR angiography showing the aneurysm (arrow) before (a) and approximately 10 min after (b) sonication.

Aneurysm creation and FUS sonications with different acoustic power and durations were carried out in five rabbits. In the first three rabbits, which were sonicated transdermally, no visible effect on the vessel could be detected on MR angiography. However, examination of the skin underlying the aneurysm revealed minor second-degree skin burns most probably induced by standing waves at the water-skin-tissue interfaces. This was consistent with MR thermometry data showing temperature elevations only at the skin surface and not around the carotid arteries. In the two rabbits with opened skin incision, temperatures of up to 54°C could be achieved next to the aneurysm. The maximum sonication duration for the last two animals was 40 s with an acoustic power of 75 W (1,750 W cm^-2^). As a result, the shape and size of the aneurysm were noticeably changed, as shown by MR angiography after the procedure (Figure 5). An increase of acoustic power at this point was inhibited by the presumed occurrence of cavitation in the sonication path and subsequent sonication inhibition enforced by the system safety mechanisms. Sonications below 75 W did not result in temperatures above 50°C or changes of form or size of the aneurysm. The treated animals recovered well after awakening from anesthesia and did not show any morbidity correlated to the sonications.

## Discussion

Due to its capacity for image guidance and precise delivery of ablative heat, MRgFUS is especially interesting in the setting of brain diseases, for which a narrow area of functional anatomy as well as limited intraoperative visual orientation often impairs safe access to lesions. While recent clinical results of MRgFUS in functional neurosurgery for tremor and chronic pain are highly encouraging [2,3], possible extension of treatment options within the spectrum of brain diseases is desirable. In this respect, an implementation of FUS on structures other than parenchymal tissue has to be considered. In terms of vascular diseases, different reports are available in which FUS is evaluated for revascularization of thrombosed vessels [7]. Furthermore, in animal studies, the application of FUS was also shown for bleeding control in injured arteries or vessel occlusion [5,4], making FUS a promising procedure to be tested in the treatment of arteriovenous malformations (AVM), cavernomas, or aneurysms. Hynynen et al. [4] were able to occlude a branch of a renal artery in rabbits (approximately 0.6 mm in diameter and 2 cm in depth from the skin surface) using a small transducer of 100-mm diameter operating at 1.5 MHz with peak intensities of up to 6,500 W cm^-2^. The device used here is optimized for patient treatments at 650 kHz; therefore, variations of animal positioning and sonication scheme were limited. Accordingly, the maximum sonication intensity we could reach was 1,750 W cm^-2^. One issue when applying FUS in the vicinity of high-perfusion areas is the heat convection by permanent blood flow. Therefore, higher acoustic power will be needed to reach ablative temperatures. Along with the increase of acoustic power comes the elevated risk of inducing cavitation, which can result in unintended tissue damage. In view of the issues mentioned, a drawback of the presented model is the short distance between the aneurysm and the skin, so that standing waves and burns are possible. In addition, the proximity to the air-filled trachea may promote cavitation formation. An alternative to add variability to the sonication scheme is the injection of microbubbles (ultrasonographic contrast agents) into the vascular system, which may enhance the efficacy of the focused ultrasound [8]. By removing the skin from the sonication path, we were able to achieve ablative temperatures, and changes in shape and size of the aneurysms were detectable. Whether this was due to temporary vasospasm, denaturation of the vessel wall, or a combination of effects could not yet be assessed. Currently, additional experiments including digital subtraction angiography follow-up and histological analysis of the vessel wall are ongoing. Taking into account the procedural difficulties encountered, further studies will use larger rabbits (approximately 1-year-old rabbits weighing 5–6 kg) and adapt the surgical method (transposition by abdominal fat pads) in order to have more tissue separating the skin and the aneurysm. Moreover, we will aim at altering the sonication effect by applying microbubbles.

## Conclusions

By slowly and moderately increasing thermal energy using MRgFUS, we could show that—despite the heat convection through high blood flow—it is possible to reach ablative temperatures at the aneurysm and to modify its shape without inducing rupture—this being a possible risk brought on by the mechanical effect of ultrasonic beam. Even if aneurysm modification with FUS may not be viable in the near future, it is important to investigate possible interferences, e.g., for the feasibility and risk assessment of patients harboring an aneurysm near a lesion to be treated with FUS. Given the relatively few animal models for larger vascular lesions such as AVM or cavernomas, the presented model offers valuable options to acquire data on the effect of FUS on larger vascular structures.

## Abbreviations

MRgFUS: MR-guided focused ultrasound; FUS: focused ultrasound; CCA: common carotid artery; VP: venous pouch; an: aneurysm; tr: trachea; sc: spinal cord.

## Competing interests

The authors declare that they have no competing interests.

## Authors’ contributions

DC carried out the surgical procedures in animals and was responsible for planning and conduction of the experiments, data analysis, and writing of the manuscript. JF was responsible for the study concept and planning of the treatment and revision of the manuscript. SM, SE, and JK contributed to the surgical procedures in animals. EM contributed to the planning of the experiments and revision of the manuscript. BW was responsible for planning and conduction of the experiments, the overall technical setup, the data acquisition and analysis and writing of the manuscript. All authors read and approved the final manuscript.
